# MUC1 Predicts Colorectal Cancer Metastasis: A Systematic Review and Meta-Analysis of Case Controlled Studies

**DOI:** 10.1371/journal.pone.0138049

**Published:** 2015-09-14

**Authors:** Yunhui Zeng, Qiongwen Zhang, Yujie Zhang, Minxun Lu, Yang Liu, Tianying Zheng, Shijian Feng, Meiqin Hao, Huashan Shi

**Affiliations:** 1 State Key Laboratory of Biotherapy, West China Hospital, West China Medical School, Sichuan University, Chengdu, Sichuan, People’s Republic of China; 2 State Key Laboratory of Biotherapy and Cancer Center, West China Hospital, West China Medical School, Sichuan University, Chengdu, Sichuan, People’s Republic of China; 3 State Key Laboratory of Biotherapy and Department of Head and Neck Oncology, West China Hospital, West China Medical School, Sichuan University, Chengdu, Sichuan, People’s Republic of China; 4 The Second Integrated Unit, West China Hospital, West China Medical School, Sichuan University, Chengdu, Sichuan, People’s Republic of China; University of Nebraska Medical Center, UNITED STATES

## Abstract

**Objective:**

To evaluate the predicting value of MUC1 expression in lymph node and distant metastasis of colorectal cancer (CRC).

**Methods:**

Pubmed/ MEDLINE and EMBASE were searched to identify eligible studies that evaluated the correlation between MUC1 and CRC. A meta-analysis was conducted to evaluate the impact of MUC1 expression on CRC metastasis.

**Results:**

A total of 18 studies (n = 3271) met inclusion criteria and the mean Newcastle-Ottawa Scale (NOS) score was 6.3 with a range from 4 to 8. The pooled OR in the meta-analysis of 15 studies indicated that positive MUC1 expression correlated with more CRC node metastasis (OR = 2.32, 95% CI = 1.63–3.29). The data synthesis of 6 studies suggested that MUC1 expression predicted more possibility of CRC distant metastasis (OR = 2.22, 95% CI = 1.23–4.00). In addition, the combined OR of 7 studies showed that MUC1 expression indicated higher Duke’s stage (OR = 3.02, 95% CI = 2.11–4.33). No publication bias was found in the mate-analysis by Begg’s test or Egger’s test with the exception of the meta-analysis of MUC1 with CRC node metastasis (Begg’s test p = 0.729, Egger’s test p = 0.000).

**Conclusions:**

Despite of some modest bias, the pooled evidence suggested that MUC1 expression was significantly correlated with CRC metastasis.

## Introduction

Mucin 1 (MUC1) is a structural transmembrane protein with a heavily glycosylated extracellular domain, which is also known as episialin, CA5-3, DF3, PAS-O, PEM, H23Ag, EMA and MCA [1,2]. MUC1 is normally expressed on the apical borders of various glandular and luminal epithelial cells in the mammary gland, esophagus, stomach, duodenum, pancreas, uterus, prostate and lungs, providing protections to the underlying epithelia and playing a role in cell signaling [3–5]. However, both distribution and biochemical features of MUC1 in cancer cells are different from those in normal cells. MCU1 is found over expressed in most human cancers and distributed over the cell surface and within the cytoplasm due to the loss of cell polarity [6]. The functional role of MUC1 in malignancy has been widely studied and several lines of evidence suggest that MUC1 is potentially correlated with the development, invasiveness and metastasis of cancer [7–14]. Meanwhile, contrary effects of MUC1 in cancer cells were reported in several independent studies [15,16]. The role of MUC1 in cancer seems to be controversial and has not been clearly clarified so far.

Colorectal cancer (CRC) is one of the most frequently diagnosed cancers and it is estimated to be the fourth most common cause of cancer death [17]. Every year, more than 1.2 million patients are diagnosed with colorectal cancer and more than 600 000 die from the disease [18]. Metastasis of cancer is correlated closely with poor prognosis and usually indicates a late stage of cancer. The five-year survival rate of CRC patients with positive regional lymph nodes or distant metastasis was significantly lower than that of patients without metastasis [19]. The over expression of MUC1 in CRC was described in many studies, as well as the potential relationship between MUC1 and metastasis. Some studies indicated MUC1 expression was positively correlated with CRC metastasis while others did not [20–30]. So far, it is not clear whether the correlation between MUC1 expression and CRC metastasis is of statistic significance, and the predictive value of MUC1 expression in CRC metastasis has not been evaluated systemically.

This systemic review and meta-analysis was designed to clarify the role of MUC1 in CRC metastasis and evaluate the correlation of MUC1 expression with node metastasis, distant metastasis and Duke’s stage of CRC.

## Methods

### Identification and Selection of Studies

According to a prespecified written protocol, the literature research was designed to identify available studies that evaluated the correlation between MUC1 expression and CRC metastasis. Specifically, CRC metastasis included node metastasis and distant metastasis. Additionally, Duke’s stage was chosen to estimate metastasis status because it was widely used in the diagnosis of CRC and could be easily divided into two categories according to metastasis or not. Duke’s stage C/D indicated node and/or distant metastasis while stage A/B indicated no metastasis. Therefore, several inclusion criteria were used to select eligible studies: (1)Patients with pathological diagnosis of colorectal cancer, regardless of pathological classification; (2) MUC1 expression in tumor tissue evaluated by immunohistochemistry (IHC) method with monoclonal antibody against MUC1; (3) Sufficient data to estimate the odd ratio (OR) and corresponding 95% Confidence Interval (CI) of the correlation of MUC1 expression with node metastasis, distant metastasis and/or Duke’s stage; (4) Case controlled studies. The excluded criteria included: (1) Patients diagnosed with recurrent CRC; (2) Full-text published in other languages rather than English or Chinese because of resource limitation.

Studies were identified by searching the electronic databases of Pubmed/MEDLINE (1950 to December 31, 2014) and EMBASE (1980 to December 31, 2014). The keywords “MUC1”, “mucin 1”, “episialin”,“CA5-3”, “DF3”, “PAS-O”, “PEM”, “H23Ag”,“EMA”, “MCA”, “colorectal cancer”, “colorectal carcinoma”, “colon cancer” and “colon carcinoma” were used in various combinations. Published language was limited to English and Chinese because of time and resources limitations. The reference lists of identified studies were also searched manually as a complement the computer searches.

Two independent reviewers screened the abstracts of primary identified studies and related references for eligibility. Full-text articles were read for further assessment if the eligibility was unclear by screening the abstracts. Discrepancies in the inclusion were resolved by discussion within the review team. For primary included studies, names of all authors and the medical centers involved were examined carefully to avoid repetitive data. Whenever studies pertained to overlapping patients, studies with larger sample size and more comprehensive data were retained.

### Data Extraction

Two independent reviewers extracted the details of included studies with a standardized form. The following information was recorded: first author, year of publication, country of origin, sample size, number of male included, mean or median age, cancer characteristics, number of patients with node metastasis, number of patients with distant metastasis; number of patients with Duke’s stage C/D; antibody used; cut-off value of positive MUC1 expression and conclusions. The conclusions of each study were termed “positive” when positive MUC1 expression predicted more node metastasis, distant metastasis or Duke’s stage C/D, “negative” when positive MUC1 expression predicted less metastasis or Duke’s stage A/B, and “indeterminate” when no significant correlation between MUC1 expression and metastasis or Duke’s stage was found. Any discrepancy was resolved through discussion. The main outcome was the number of patients with node metastasis, distant metastasis or Duke’s stage C/D in MUC1 positive and negative group.

### Quality Assessment

According to the Cochrane Collaboration, the Newcastle-Ottawa Scale (NOS) was used to assess the quality of the included studies by judging on three board perspectives: the selection of study groups, the comparability of study groups and the measurement of exposure in study groups [31, 32].

### Data Synthesis

The included studies were divided into three groups for analysis: those with data regarding node metastasis, those with data regarding distant metastasis and those with data regarding Duke’s stage. The correlation of MUC1 with node metastasis, distant metastasis and Duke’s stage in each study was estimated by OR and corresponding 95% CI. Heterogeneity chi-squared (Χ^2^)and I-squared (I^2^) value were computed to assess the heterogeneity of the included studies in each group. If significant heterogeneity was found i.e. P< = 0.10 and/or I^2^>50%, potential sources of heterogeneity between studies would be explored. If the heterogeneity could not be eliminated by subgroup analysis, the ORs of each group were combined respectively in a random effect meta-analysis using DerSimonian-Laird algorithm. If the heterogeneity was acceptable, Mantel-Haenszel algorithm was used in a fixed effect model. Meta-analyses were carried out with the Stata version 11.0 (Stata Corporation, College Station, TX, USA). The pooled OR>1 indicated more metastasis and Duke’s stage C/D in MUC1 positive group relative to MUC1 negative group, which would be considered significant if the 95%CI did not overlap 1, with p<0.05.

### Subgroup and Sensitivity Analyses

Based on prespecified protocol of the meta-analysis, sub-group analysis was performed according to the monoclonal antibody against MUC1 and sensitivity analysis was performed when limiting the cut-off value of positive MUC1 expression. The effect of potential publication bias on results was assessed by Begg’s test and Egger’s test [33, 34].

## Results

### Studies Selection and Characteristics

**Fig 1:**
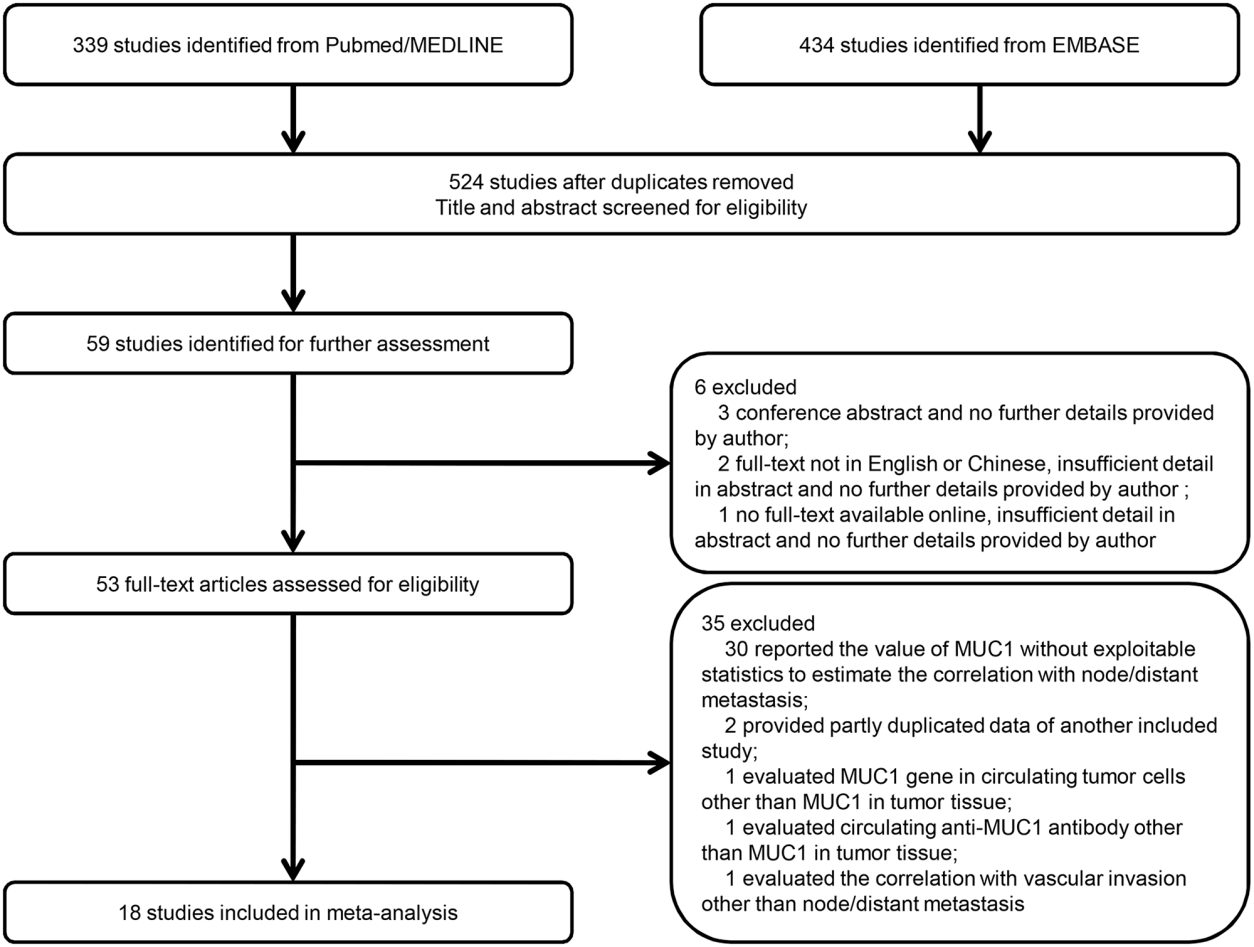
Flow diagram showing inclusion and exclusion of studies.

A total of 524 studies were initially identified after duplications removed through the prespecified search strategy, and 59 studies were retrieved for full-text following abstract screening. There were 3 conference abstracts, 2 studies not in English or Chinese and 1 study without full-text available online despite of any effort, and therefore they were excluded [35]. Full-texts of 53 studies were assessed carefully and 35 studies were excluded for several reasons as shown in Fig 1 [22, 36–39]. Finally, a total of 18 studies (n = 3271) met all the inclusion criteria and were included in meta-analysis [20,21,23–30,40–47].

The characteristics of the included studies were provided in Table 1. The 18 included studies were published between 1988 and 2014, and the original countries were distributed in all continents except for Africa. The median sample size of all studies was 91.5 patients (range = 31–1414) and the mean proportion of male patients was about 55.0%. The median age of patients in 15 studies was 64.12 years old (range = 56.2–71). In all the studies, MUC1 expression was evaluated by IHC staining in formalin-fixed paraffin-embedded tissue blocks using several monoclonal antibodies against MUC1 including Ma695, Ma552, KL-6, HMFG-1, HMFG-2, ICR2, 139H2 and MUSE11. The cut-off value of MUC1 positive or negative was mostly estimated by the proportion or area of positive stained cell in tumor tissue that ranged from 0 to 35%, while 4 studies did not reported the exact definition of MUC1 positive. The most common used cut-off value was> = 30% tumor cells stained positive (N = 5). The correlation of MUC1 expression with metastasis was positive in 11 studies and indeterminate in the other 7 studies. The relationship between MUC1 expression and CRC node metastasis could be estimated in 15 of the 19 included studies, while the correlation of MUC1 expression with CRC distant metastasis and Duke’s stage could be estimated in 6 and 7 studies respectively.

**Table 1:** Main characteristics of the 18 included studies.

Ref.	First Author	Year	Country	n(Male)	Median Age (y)	Patients with Node Metastasis	Patients with Distant Metastasis	Patients with Duke’s Stage C/D	Antibody	Cut-off Value of MUC1 Positive	OR Estimation	Conclusions
20	**Ajioka Y**	**1996**	**Australia**	**51(23)**	**?**	**24**	**?**	**?**	**MUSE11**	**> = 30**	**Data extrapolated**	**Indeterminate**
21	**Baldus SE**	**2004**	**Germany**	**205(102)**	**65.57**	**99**	**?**	**?**	**HMFG-2**	**> = 35%**	**Data extrapolated**	**Positive**
40	**Baldus SE**	**2002**	**Germany**	**243(121)**	**65.5**	**126**	**18**	**?**	**HMFG-2**	**> 35%**	**Data extrapolated**	**Positive**
41	**Davidson BR**	**1988**	**UK**	**31(?)**	**?**	**6**	**7**	**?**	**ICR2**	**Positive**	**Data extrapolated**	**Indeterminate**
42	**Davidson BR**	**1989**	**UK**	**51(26)**	**68.00**	**20**	**?**	**20**	**ICR2**	**Positive**	**Data extrapolated**	**Indeterminate**
43	**Guo Q**	**2006**	**Japan**	**82(55)**	**64.5**	**36**	**7**	**?**	**KL-6**	**Positive**	**Data extrapolated**	**Positive**
23	**Huang WB**	**2002**	**China**	**126(74)**	**56.2**	**70**	**?**	**76**	**Ma695**	**> = 30%**	**Data extrapolated**	**Positive**
24	**Jang KT**	**2002**	**Korean**	**97(53)**	**65**	**44**	**?**	**?**	**Ma552**	**> = 30%**	**Data extrapolated**	**Positive**
44	**Kaneko I**	**2007**	**Japan**	**214(?)**	**?**	**28**	**?**	**?**	**KL-6**	**> = 10%**	**Data extrapolated**	**Positive**
25	**Karamitopoulou E**	**2011**	**Greece**	**212(102)**	**68.1**	**106**	**?**	**?**	**139H2**	**Positive**	**Reported**	**Positive**
26	**Kimura T**	**2000**	**Japan**	**110(69)**	**63.1**	**59**	**33**	**63**	**KL-6**	**> = 30%**	**Data extrapolated**	**Positive**
45	**Lugli A**	**2007**	**Switzerland**	**1414(673)**	**71**	**652**	**?**	**?**	**139H2**	**> 0%**	**Data extrapolated**	**Indeterminate**
27	**Matsuda K**	**2000**	**Japan**	**86(62)**	**61.9**	**36**	**6**	**36**	**Ma695**	**> = 30%**	**Data extrapolated**	**Positive**
46	**Mulder WMC**	**1996**	**Netherlands**	**56(?)**	**?**	**19**	**?**	**19**	**HMFG-1**	**> = 5%**	**Data extrapolated**	**Indeterminate**
47	**Oshima T**	**2007**	**Japan**	**63(34)**	**65**	**48**	**23**	**48**	**Ma695**	**> = 10%**	**Data extrapolated**	**Indeterminate**
28	**Perez RO**	**2008**	**Brazil**	**35(20)**	**62.2**	**14**	**?**	**?**	**Ma695**	**> 10%**	**Data extrapolated**	**Indeterminate**
29	**Yu XW**	**2007**	**China**	**150(95)**	**57.5**	**72**	**?**	**72**	**Ma695**	**> 5%**	**Data extrapolated**	**Positive**
30	**Zhang XW**	**2014**	**China**	**45(26)**	**?**	**19**	**?**	**?**	**ZM-0391**	**> 25**	**Data extrapolated**	**Positive**

### Quality Assessment of Included Studies

The qualities of included studies assessed with NOS were provided in Table 2. The mean total score was 6.3 with a range from 4 to 8. The selection of patients with pathological confirmed cases was appropriate and representative in most of studies. The comparability between case group and control group was limited since confounding factors such as age, sex, location and subtype of cancer were not controlled sufficiently. MUC1 expression was identified by IHC with different antibodies in all studies while non-response rate of each group was not reported or compared.

**Table 2:** Quality assessment of the 18 included studies with the Newcastle-Ottawa Scale (NOS).

Ref.	First Author	Year	Selection of subjects/4	Comparability of groups/2	Measurement of Exposure/3	Total score of NOS/9
20	Ajioka Y	1996	3	1	2	6
40	Baldus SE	2002	4	1	2	7
21	Baldus SE	2004	4	1	2	7
41	Davidson BR	1988	2	1	2	5
42	Davidson BR	1989	3	1	2	6
43	Guo Q	2006	4	1	2	7
23	Huang WB	2002	3	1	2	6
24	Jang KT	2002	4	1	2	7
44	Kaneko I	2007	3	1	2	6
25	Karamitopoulou E	2011	3	1	2	6
26	Kimura T	2000	4	1	2	7
45	Lugli A	2007	4	2	2	8
27	Matsuda K	2000	3	1	2	6
46	Mulder WMC	1996	2	0	2	4
47	Oshima T	2007	3	1	2	6
28	Perez RO	2008	3	1	2	6
29	Yu XW	2007	4	1	2	7
30	Zhang XW	2014	3	1	2	6

### MUC1 and CRC Node Metastasis

**Fig 2:**
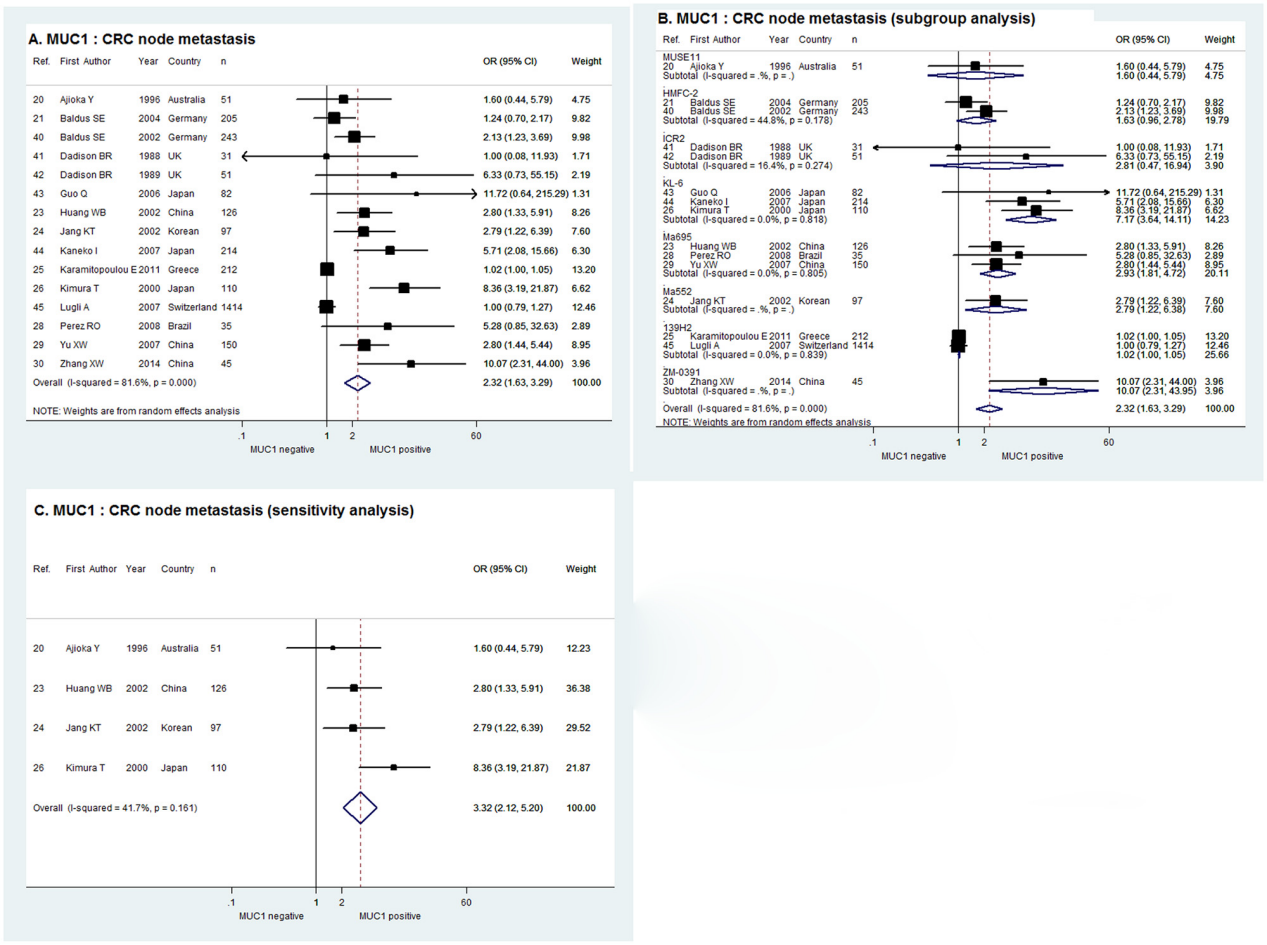
Forrest plots of meta-analysis of studies evaluating the correlation of MUC1 expression with CRC node metastasis. Overall meta-analysis (A), subgroup analysis according to antibodies used (B) and sensitivity analysis when limiting the cut-off value to > = 30% tumor cells stained positive (C).

As shown in Fig 2A, the pooled OR in random effect meta-analysis of the 15 studies evaluating the correlation of MUC1 expression with CRC node metastasis was 2.32with the corresponding 95% CI of 1.63–3.29 (χ^2^ = 75.92, P = 0.000, I^2^ = 81.6%). In the sub-group analysis according to the monoclonal antibody against MUC1, a positive relationship between MUC1 expression and node metastasis was demonstrated by the combined OR of the 3 studies using KL-6 (OR = 7.17, 95% CI = 3.64–14.11), and that of the 3 studies using Ma695 (OR = 2.93, 95% CI = 1.81–4.72) and the 2 studies using 139H2 (OR = 1.02, 95% CI = 1.00–1.05). Meanwhile, the combined ORs of the studies using HMFC-2 and ICR2 were indeterminate with 95% CIs overlapping 1 and there was only one study using MUSE11, Ma552 and ZM-0391 respectively (Fig 2B). When limiting the cut-off value to> = 30% tumor cells stained positive, the sensitivity analysis of the 4 studies in fixed effect model indicated that MUC1 expression was significantly correlated with CRC node metastasis with combined OR of 3.32 and 95% CI of 2.12–5.20 (χ^2^ = 5.15, P = 0.161, I^2^ = 41.7%,Fig 2C).

### MUC1 and CRC Distant Metastasis

**Fig 3:**
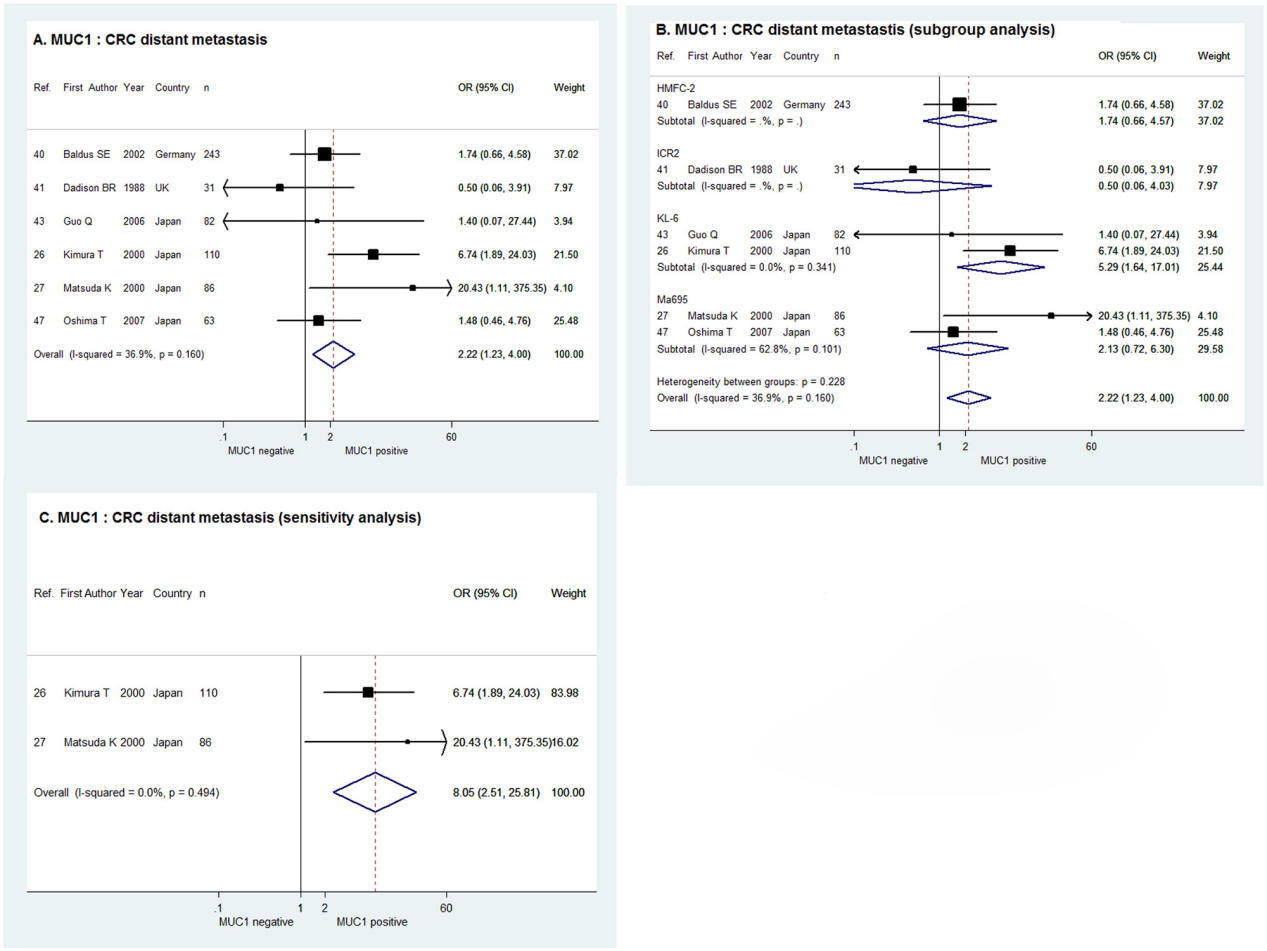
Forrest plots of meta-analysis of studies evaluating the correlation of MUC1 expression with CRC distant metastasis. Overall meta-analysis (A), subgroup analysis according to antibodies used (B) and sensitivity analysis when limiting the cut-off value to > = 30% tumor cells stained positive (C).

For the 6 studies estimating the correlation between MUC1 expression and CRC distant metastasis, the combination of ORs in fixed effect meta-analysis suggested that distant metastasis of CRC was more likely to occur when MUC1 expression was positive (OR = 2.22, 95% CI = 1.23–4.00, χ^2^ = 7.92, P = 0.160, I^2^ = 36.9%), as was shown in Fig 3A. The sub-group analysis by antibody used demonstrated a positive correlation between MUC1 expression and CRC distant metastasis in the 2 studies using KL-6 (OR = 5.29, 95% CI = 1.64–17.01), whereas the correlation was indeterminate in studies using HMFC-7, ICR2 and Ma695 (Fig 3B). For the 2 studies with cut-off value of > = 30%, the sensitivity analysis in fixed effect model showed positive MUC1 expression indicated more CRC distant metastasis with the combined OR of 8.05 and 95% CI of 2.51–25.81 (χ^2^ = 0.47, P = 0.494, I^2^ = 0.0%, Fig 3C).

### MUC1 and CRC Duke’s Stage

**Fig 4:**
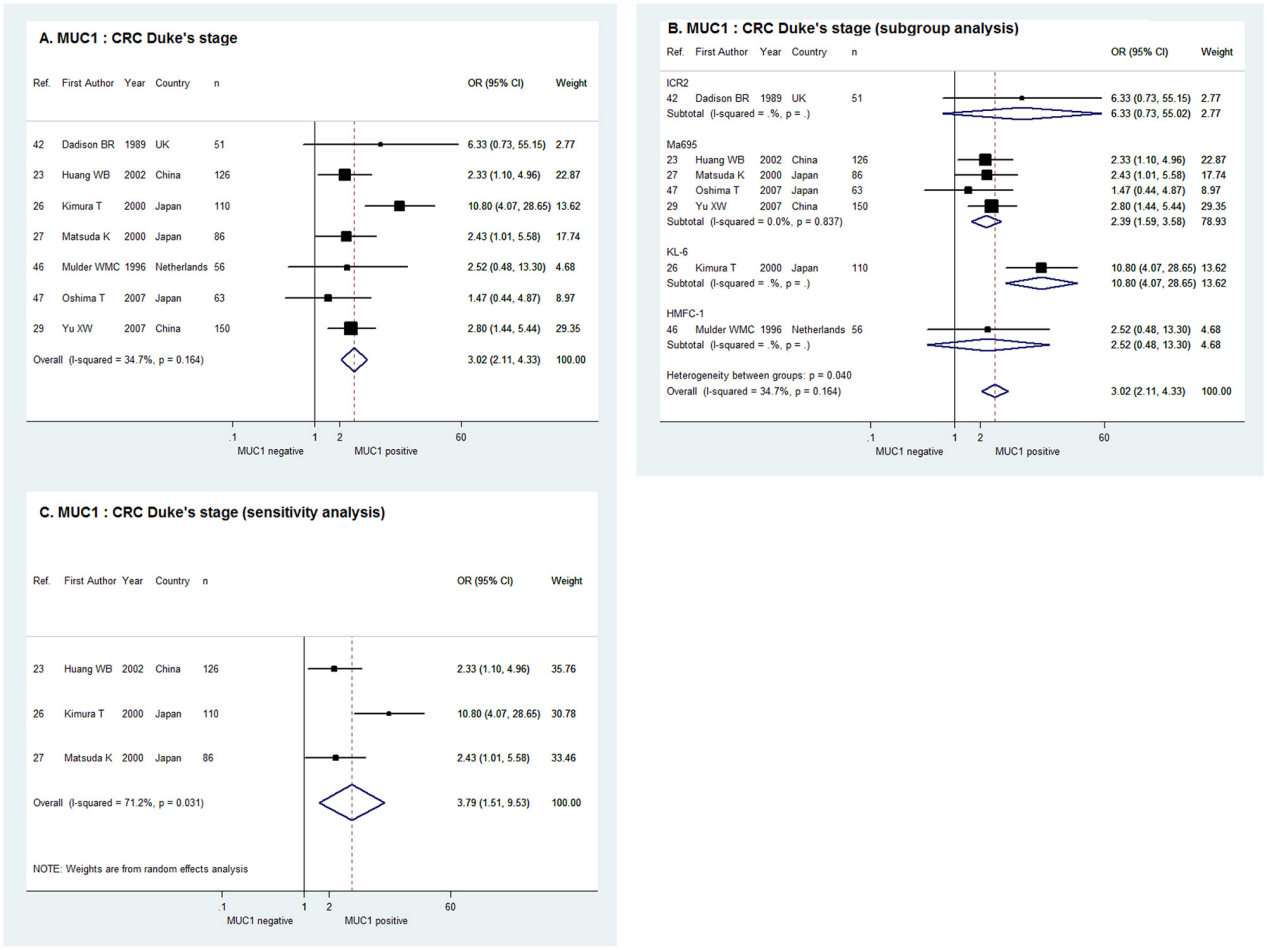
Forrest plots of meta-analysis of studies evaluating the correlation of MUC1 expression with CRC Duke’s stage. Overall meta-analysis (A), subgroup analysis according to antibodies used (B) and sensitivity analysis when limiting the cut-off value to > = 30% tumor cells stained positive (C).

As shown in Fig 4A, the fixed effect meta-analysis of the 7 studies indicated a positive relationship between MUC1 expression and CRC Duke’s stage with the pooled OR of 3.02 and 95% CI of 2.11–4.33 (χ^2^ = 9.18, P = 0.164, I^2^ = 34.7%). In the sub-group analysis according to the antibody against MUC1, the combined OR of the 4 studies usingMa695 (OR = 2.39, 95% CI, 1.59–3.58) and the OR of the single studyusingKL-6 (OR = 10.80, 95% CI = 4.07–28.65) confirmed the correlation of MUC1 expression with Duke’s stage C/D. However, the ORs of the single study usingICR2 and HMFC-1 were indeterminate with 95% CIs overlapping 1 (Fig 4B). For the 3 studies with cut-off value of > = 30%, the sensitivity analysis in random effect model demonstrated positive MUC1 expression was significantly correlated with higher Duke’s stage of CRC with the combined OR of 3.79 and 95% CI of 1.51–9.53 (χ^2^ = 6.95, P = 0.031, I^2^ = 71.2%, Fig 4C).

### Evaluation of Publication Bias

**Fig 5:**
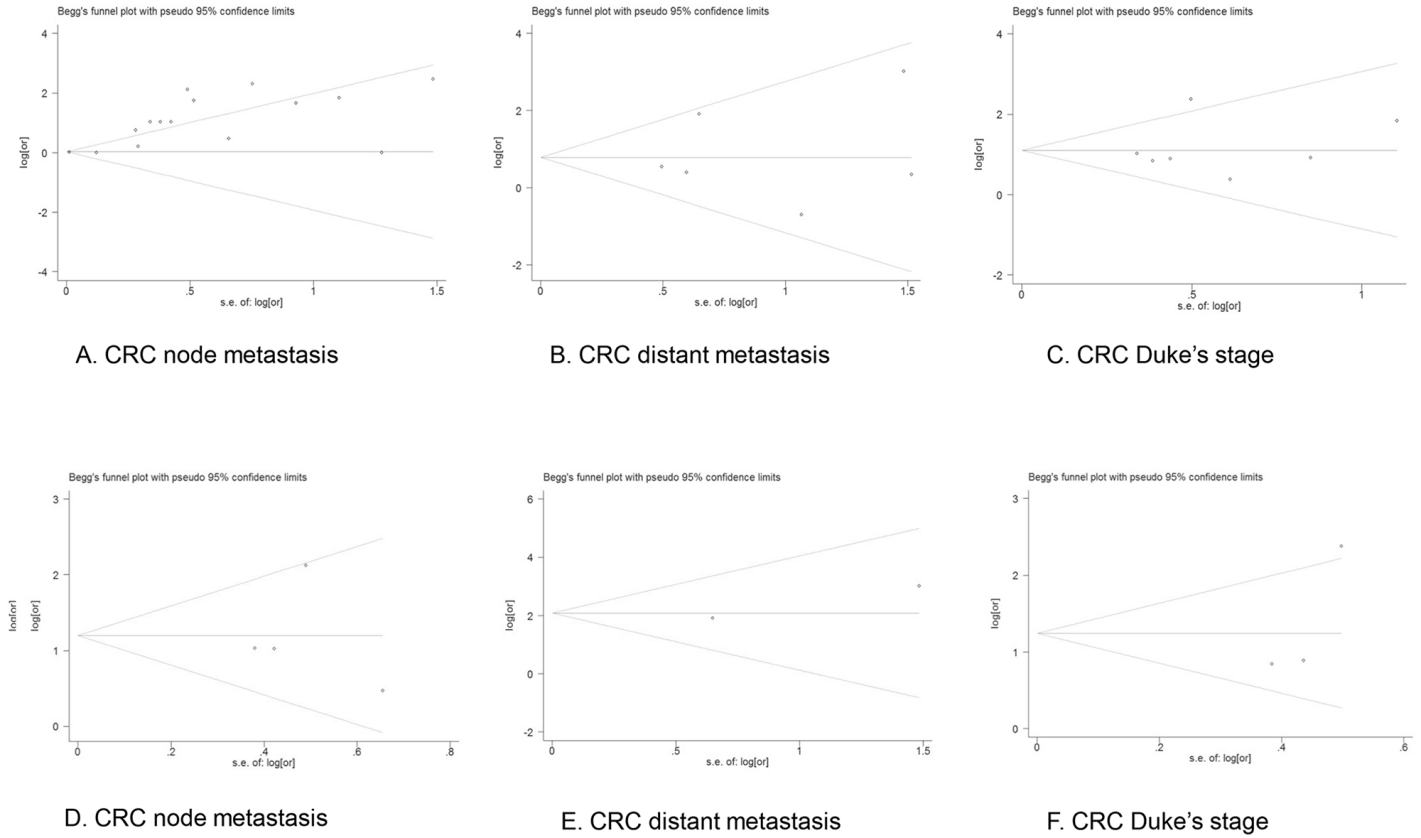
Funnel plot of Begg’s test of meta-analysis (A-C) and sensitivity analysis (D-F). Funnel plot of the meta-analysis of CRC node metastasis (A), distant metastasis (B) and Duke’s stage (C) in Begg’s test, and those of sensitivity analysis of CRC node metastasis (D), distant metastasis (E) and Duke’s stage (F).

**Fig 6:**
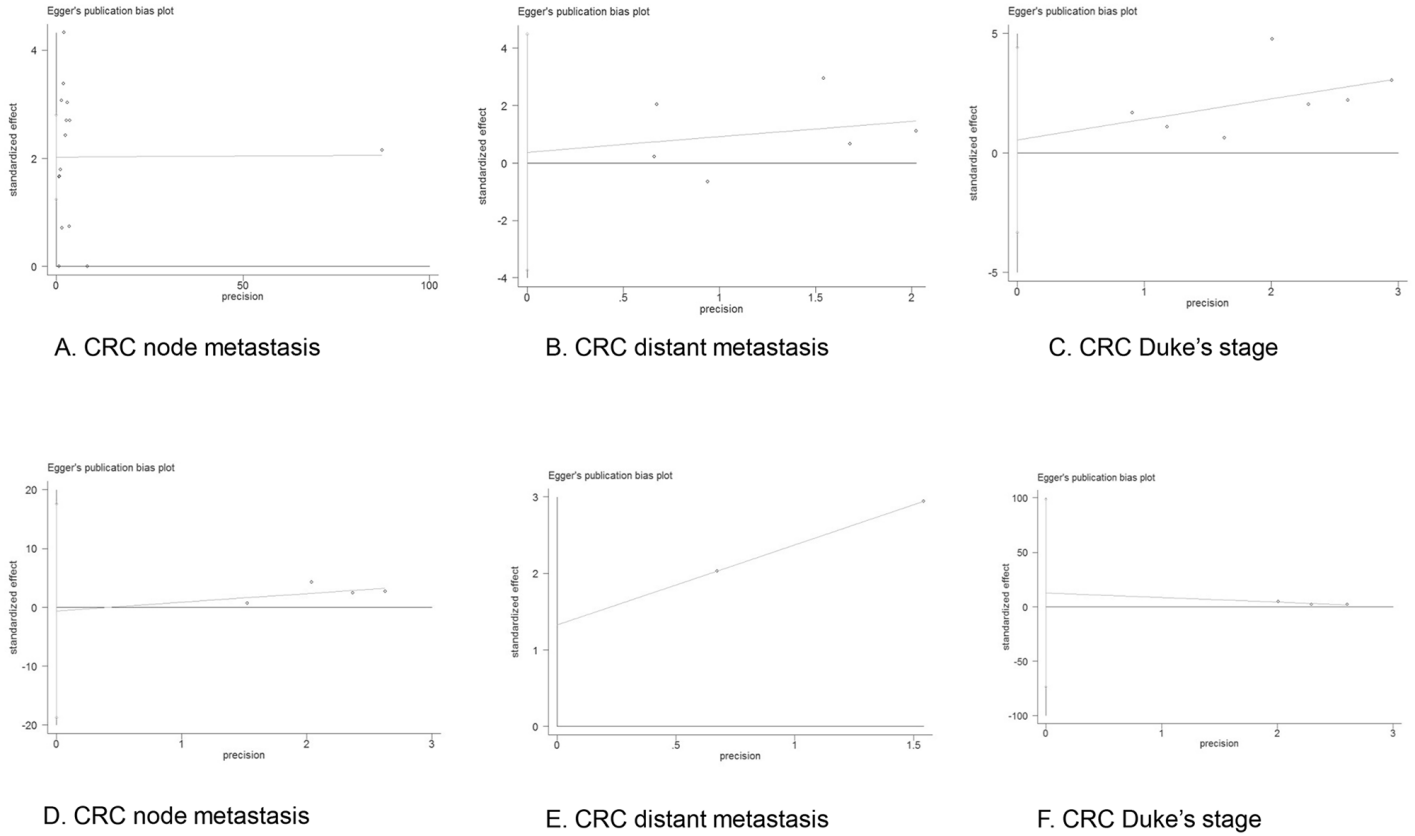
Funnel plot of Egger’s test of meta-analysis (A-C) and sensitivity analysis (D-F). Funnel plot of the meta-analysis of CRC node metastasis (A), distant metastasis (B) and Duke’s stage (C) in Egger’s test, and those of sensitivity analysis of CRC node metastasis (D), distant metastasis (E) and Duke’s stage (F).

Both Begg’s test and Egger’s test were performed to examine the potential publication bias in all the meta-analysis and sensitivity analysis. No evidence of publication bias was found by Begg’s test in the overall meta-analysis of MUC1 expression with CRC node metastasis (p = 0.729), distant metastasis (p = 0.851) or Duke’s stage (p = 0.453), while Egger’s test revealed possible publication bias in the analysis of node metastasis (p = 0.000) but no bias in the analysis of distant metastasis (p = 0.811) or Duke’s stage (p = 0.729). In addition, both Begg’s test and Egger’s test showed no publication bias in the sensitivity analysis of node metastasis (p = 1.000, p = 0.902), distant metastasis (p = 0.317, p = N/A) or Duke’s stage (p = 0.117, p = 0.310). The funnel plots of the Begg’s test and Egger’s test were shown in Figs 5 and 6.

## Discussion

Previous studies found MUC1 was usually over expressed in CRC tissue and seemed to play a role in the tumor development and progression. This was the first meta-analysis to systematically evaluate the correlation between MUC1 expression and CRC metastasis. The results revealed that positive MUC1 expression in CRC tissue was strongly correlated with more metastasis of CRC, which was consistent with the conclusions of most included studies. Present evidence from the meta-analysis indicated that MUC1 might be a promising biomarker to predict the status of CRC metastasis at the time of diagnosis. The expression of MUC1 in CRC tissue can be determined by IHC method with monoclonal antibody against MUC1, which can be done simultaneously when pathologists conduct pathological diagnosis with tumor tissue obtained from surgery or biopsy. It is relatively easy to realize and popularize in clinical practice, which is of great significance to help surgeons determine treatment strategy. The establishment of the relationship between MUC1 expression and CRC metastasis may also help to clarify the metastasis risk of CRC patients at the time of diagnosis, especially those without symptoms or signs of metastasis. If MUC1 is highly expressed in a CRC patient without metastatic manifestation, it is worthy to do more detailed examination in search for any existing small metastasis. Patients with over expressed MUC1 may need more radical or aggressive treatment after diagnosis and more rigorous care after tumor resection due to a relatively high risk of metastasis. However, present study only proved the correlation between MUC1 expression and CRC metastasis. The positive rate of MUC1 expression in CRC patients with metastasis differed greatly in previous retrospective studies, and the sensitivity and specificity of MUC1 in predicting CRC metastasis have not been validated in prospective studies. Besides, MUC1 expression in CRC tissue cannot guide therapy before surgery or biopsy. Despite of these limitations, MUC1 deserves further investigation in CRC patients.

Among the included studies in the meta-analysis, several monoclonal antibodies were used to detect the expression of MUC1. Although the expression level of MUC1 stained by different antibodies was not compared in any of the included studies, the positive rate of MUC1 expression was different among studies using different antibodies. In CRC patients with metastasis, the average positive rate was 44.6% (range from 20.0% to 62.7%) in the 6 studies using Ma695, 63.5% (range from 49.5% to 92.7%) in the 3 studies using KL-6 and 40.6% (range from 32.5% to 50.2%) in the 2 studies using HMFG-2. The difference may be attributed to many interference factors, but the various antibodies should be concerned first. Subgroup analysis was then performed according to the monoclonal antibody and the results or tendencies were basically consistent among different subgroups, which were also consistent with those of the overall meta-analysis. Together with sensitivity analysis, the results of subgroup analysis confirmed that positive MUC1 expression was correlated with more CRC metastasis in spite of the limited number of included studies.

Node metastasis, distant metastasis and Duke’s stage were used in the meta-analysis to estimate the metastasis status of CRC because they are widely applied in clinical practice and closely correlated with TNM stage and prognosis of CRC patients. Given the difference in economy and medical technology, some small metastasis might not be detected at the time of diagnosis, which would cause interference to the results of retrospective case controlled studies. However, it in turn emphasizes that it is necessary to find a reliable approach to predict the metastasis status of CRC in advance. Prospective studies are needed to avoid this interference and evaluate the relationship between MUC1 expression and CRC metastasis, as well as the sensitivity and specificity of MUC1 in predicting CRC metastasis status.

The potential role of MUC1 in malignancy has been widely investigated in recent years. Several lines of evidences have demonstrated that MUC1 expression is correlated with tumor proliferation, metabolism, invasion, metastasis, angiogenesis and resistance to apoptosis [11, 16, 48–58]. MUC1 can mediate production of several growth factors such as connective tissue growth factor (CTGF), platelet-derived growth factor A (PDGF-A) and PDGF-B, which promote the proliferation and survival of tumor cells [11, 16,48–50]. In the metabolism of cancer cells, MUC1 modulate the expression of glycolytic pathway enzymes by interacting with HIF-1αand enhances the expression of genes pertaining to glucose uptake and metabolism [51]. MUC1 can induce epithelial to mesenchymal transition (EMT) by upregulating the expression of inducers Snail, Slug, Vimentin, and Twist, as well as by modulating the expression of miRNAs which control the expression of EMT-related genes [52,53]. Many studies have suggested MUC1 expression is correlated with metastasis and one of the possible mechanisms is that MUC1 acts as a ligand for cell adhesion molecules and help MUC1-expressing circulating tumor cells (CTCs) adhere to endothelial cells and seed at distant site to establish secondary tumors [54]. The metastatic role of MUC1 was also attributed to the interaction of MUC1 and platelet-derived growth factor receptor β, as well as the transcriptional regulations of MUC1 on CTGF [11, 49]. Angiogenesis is essential during the development of tumor and multiple proangiogenic factors such as vascular endothelial growth factor-A (VEGF-A) and PDGF-B can be induced by MUC1 under hypoxia, which promote the synthesis of new blood vessels within the tumor masses [55]. It has been reported that MUC1 can enhance the expression of anti-apoptotic protein, inactivate the pro-apoptotic protein, decrease the intracellular reactive oxygen species (ROS) levels and upregulate the expression of multidrug resistance gene and protein, resulting in cancer cell resistant to apoptosis and chemotherapeutic drugs [56–58]. Based on these findings, MUC1 has been applied to clinical practice as a cancer biomarker for diagnosing, staging and monitoring relapse following therapy [59, 60]. MUC1 has also been considered as a target to develop MUC1-based immunotherapy, which may benefit CRC patients with high expression of MUC1 by reducing the risk of metastasis and prolonging the survival [61–63].

Although a vast majority of studies indicated the proliferative and metastatic role of MUC1, several studies did indicate anti-proliferative and anti-metastatic effect of MUC1. Downregulation of MUC1 expression was found to increase proliferation and apoptosis in MKN45 gastric carcinoma cell line, but *in vivo* study showed that mice injected with MUC1 downregulated cells developed smaller tumors when compared to those injected with the control cells [15]. In another study, MUC1 siRNA in MDA-MB-468 breast cancer cell line was reported to decrease proliferation and invasion and increase stress-induced apoptosis, but MUC1 siRNA in BT-20 breast cancer cell line increased proliferation [16]. Furthermore, in a study with S2-013.MUC1F pancreatic cancer cells, MUC1 expression conferred on tumors a greater propensity to metastasize when present in low hepatocyte growth factor (HGF) tissue environments but conversely downregulated HGF-stimulated activation of motility and invasion under conditions of high HGF concentrations [64]. The contradiction may be partially explained by the difference of individual cell lines or cellular microenvironments, but the context-dependent functions of MUC1 in malignancy seem to be more multifaceted than previous comprehension. Our meta-analysis clarified that positive MUC1 expression was strongly correlated with more CRC metastasis, which suggested that MUC1 exerted proliferative and metastatic effect in CRC.

Although the systematic review and meta-analysis was conducted strictly in accordance with the Cochrane Collaboration, several limitations and potential bias should be taken into consideration when the results were interpreted. First of all, the most common pathological classification of CRC was adenocarcinoma with various differentiation degrees in the included studies, the correlation of MUC1 expression with other pathological types or certain differentiation degree was not evaluated sufficiently. As the biological properties of different types of CRC varied from each other, the results of this meta-analysis could not present the actual impact of MUC1 expression on certain subtype of CRC. However, a high qualified case controlled study with a large sample size explored the effect of MUC1 expression in CRC patients with different DNA mismatch-repair (MMR) status [45]. The result suggested a positive correlation or tendency of MUC1 expression with node metastasis of MMR-proficient CRC (OR = 1.06, 95% CI = 0.82–1.34), MLH1-negative CRC (OR = 1.02, 95% CI = 0.44–2.33) and presumed hereditary non-polyposis colon cancer (HNPCC) (OR = 0.81, 95% CI = 0.23–2.82), which was almost consistent with the impact of MUC1 expression on the node metastasis of overall CRC (OR = 1.00, 95% CI = 0.79–1.27) in the study. Further studies are needed to evaluate the different significance of MUC1 in various subtypes of CRC.

Secondly, the NOS scores of the included studies ranged from 4 to 8 with a mean score of 6.3, suggesting the qualities were just acceptable but unsatisfactory. Based on the NOS scores, the main limitations were focused on the comparability between groups and non-response rate. The difference in confounding factors, such as age, sex, location and subtype of cancer, were not reported or compared between case group and control group, which might weaken the reliability of the results. Given that MUC1 expression was identified by researchers with experimental methods and was different from other exposure factors such as nicotine and alcohol that needed to ask patients to collect the information, the non-response rate might not necessary or applicable for the experimental studies here.

Thirdly, random effect model was used in the meta-analysis of the 15 studies involving CRC node metastasis and the sensitivity analysis of the 3 studies involving Duke’s stage due to the potential heterogeneity between studies. However, the results were consistent with those of other analyses in fixed effect model, suggesting the heterogeneity may not make great differences in the analyses. Furthermore, publication bias was identified in the meta-analysis of node metastasis (p = 0.000) by Egger’s test while no bias was found by Begg’s test, which could be largely attributed to the limited number of included studies. The limitations in sample size, antibodies used and publication language should also be considered when interpreting the results in clinical practice.

In conclusion, it is clear that MUC1 expression is significantly correlated with more CRC metastasis. Despite of some modest bias, the results of this meta-analysis has provided strong evidence that positive MUC1 expression indicates higher Duke’s stage and more possibility of node and distant metastasis in CRC patients. MUC1 can be used as a biomarker to identify the metastatic potential of CRC and also as a promising target for future immunotherapy to decrease the risk of metastasis and prolong the survival of CRC patients.

## Supporting Information

S1 File PRISMA Checklist. (DOC)

S2 File Original figures of Figs 2–6 obtained by STATA. (RAR)
